# Supplementary figures and images for: Fine‐scale coexistence between Mediterranean mesocarnivores is mediated by spatial, temporal, and trophic resource partitioning
Source: Ecol Evol. 2021 Nov 9;11(22):15520–33. doi: 10.1002/ece3.8077 (PMC8601891; doi:10.1002/ece3.8077)

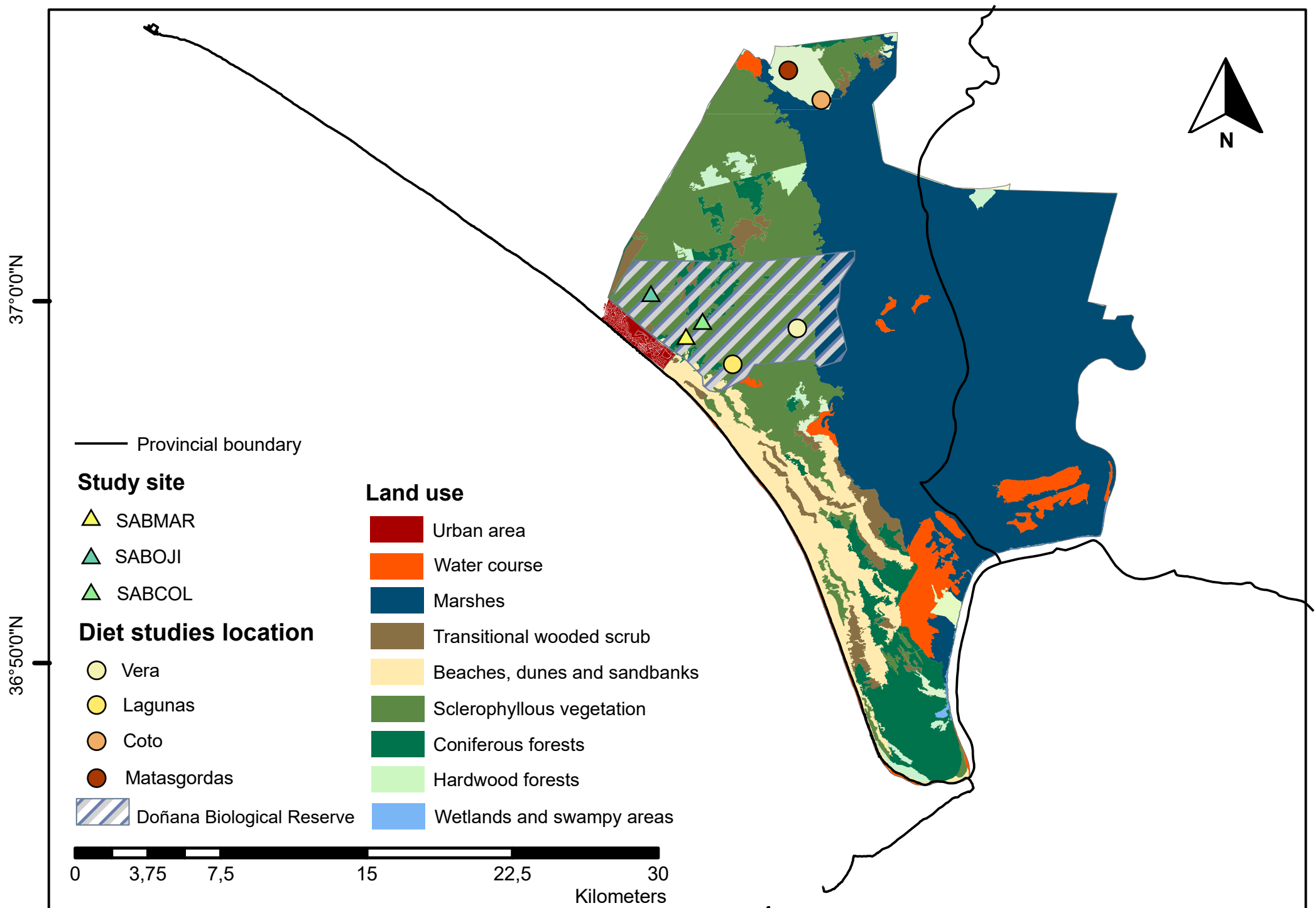

Distances from diet studies to study sites: Matasgordas, 14 km; Coto del Rey, 13,5km; Vera, 6.3km; and Lagunas, 3,7km.

Supplement: Supplementary file 1 — Figure S1 [file ECE3-11-15520-s003.pdf]

A

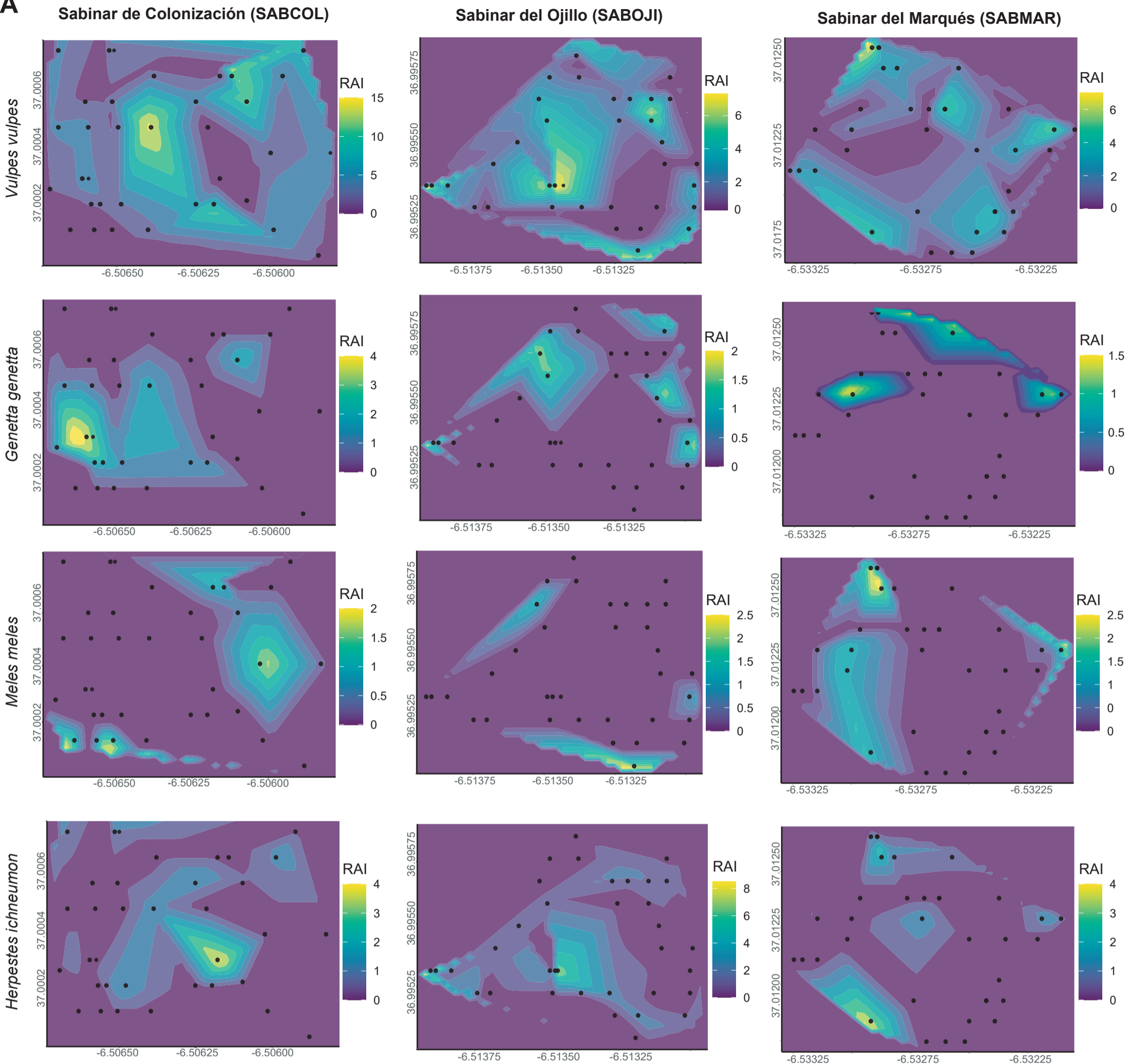

B

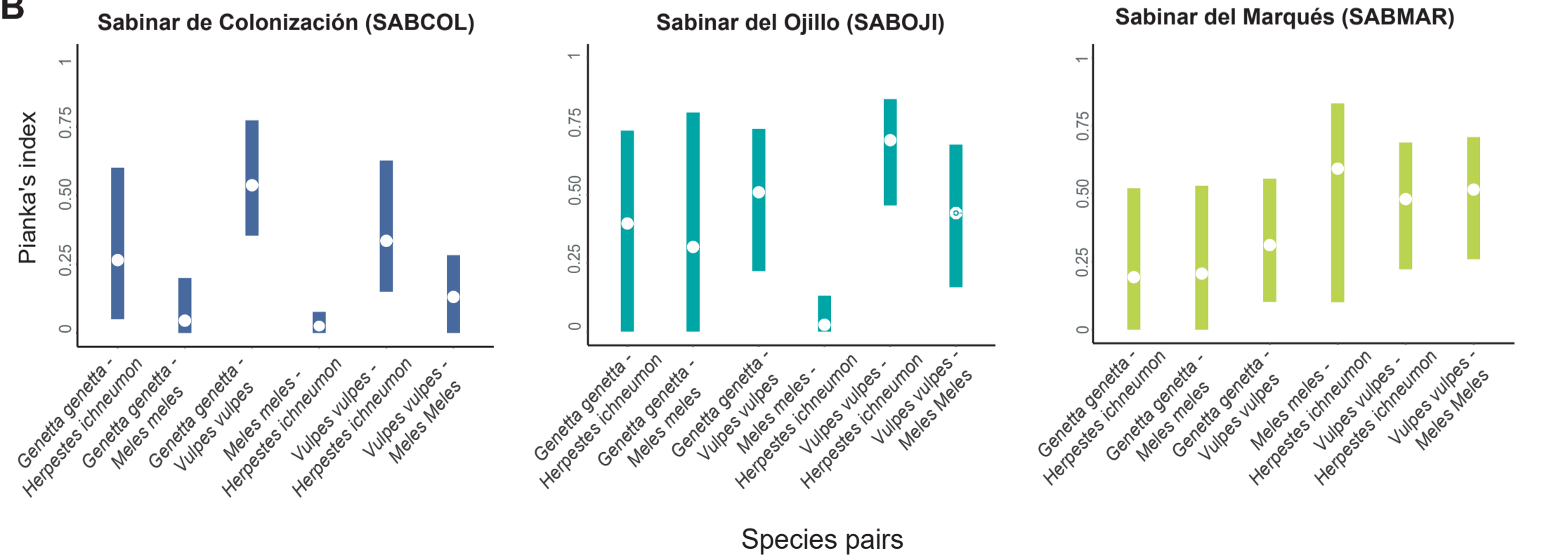

Supplement: Supplementary file 2 — Figure S2 [file ECE3-11-15520-s005.pdf]
